# Levodopa‐Entacapone‐Carbidopa Intestinal Gel Treatment in Advanced Parkinson's Disease: A Single‐Center Study of 30 Patients

**DOI:** 10.1002/mdc3.13926

**Published:** 2023-12-10

**Authors:** Vili Viljaharju, Tuomas Mertsalmi, K. Amande M. Pauls, Maija Koivu, Johanna Eerola‐Rautio, Marianne Udd, Eero Pekkonen

**Affiliations:** ^1^ Department of Neurology Helsinki University Hospital Helsinki Finland; ^2^ Department of Clinical Neurosciences (Neurology) University of Helsinki Helsinki Finland; ^3^ Department of Gastroenterological Surgery Helsinki University Hospital Helsinki Finland

**Keywords:** LECIG, advanced Parkinson's disease, levodopa‐entacapone‐carbidopa intestinal gel

## Abstract

**Background:**

Levodopa‐entacapone‐carbidopa intestinal gel (LECIG) is a novel device assisted treatment option for advanced Parkinson's disease (PD). It has been available in Finland since 2020. There is paucity of scientific studies considering LECIG treatment in clinical practice.

**Objectives:**

Objectives of this study were to evaluate the changes in medication, adverse events and early discontinuations of LECIG treatment in real life clinical practice.

**Methods:**

The records of 30 consecutive patients, who received LECIG between years 2020 and 2022 in Helsinki University Hospital, were retrospectively analyzed. Data considering changes in medication, discontinuations, and adverse events during the first six months of LECIG treatment was collected.

**Results:**

Mean levodopa equivalent daily dose (LEDD) rose significantly between baseline before LECIG and six months with treatment (1230 mg vs. 1570 mg, *P* = 0.001). Three patients were discarded during nasojejunal tube test phase and seven discontinued the treatment during six‐month follow‐up. Most common reasons for discontinuation were difficulty in finding suitable infusion rate and neuropsychiatric problems. Safety issues encountered were similar to those reported with levodopa‐carbidopa intestinal gel (LCIG) treatment. One case of rhabdomyolysis due to severe dyskinesia during LECIG treatment was observed. Patients were satisfied with the small size of the pump system.

**Conclusions:**

LEDD seems to increase during the first months of LECIG treatment. When compared to studies on LCIG, safety profile of LECIG appears similar, but early discontinuation rate is higher than expected. However, long‐term studies are lacking. Only clear advantage to LCIG appears to be the smaller LECIG pump size.

Parkinson's disease (PD) is among the most common neurodegenerative disorders.^1^ Levodopa is currently the mainstay of symptomatic treatment of PD.^2^ As the disease progresses, patients typically develop dyskinesia and motor fluctuations after 5–10 years of treatment despite optimization of oral drug therapy.^3^ To address these motor complications of advanced PD, device assisted therapies (DAT), namely deep brain stimulation (DBS), apomorphine infusion (APO) and levodopa‐carbidopa intestinal gel (LCIG), have been developed.^4^

LCIG treatment is shown to increase ON time, decrease OFF time and dyskinesia, enhance quality of life and decrease non‐motor symptoms.^5^, ^6^, ^7^, ^8^, ^9^ It has been in use for more than 20 years in multiple different countries.^6^ Levodopa‐entacapone‐carbidopa intestinal gel (LECIG) (Britannia Pharmaceuticals Ltd) is a novel DAT option for advanced PD.^10^ Compared to LCIG, LECIG contains catechol‐O‐methyltransferase (COMT) inhibitor entacapone (20 mg/ml), in addition to levodopa (20 mg/ml) and carbidopa (5 mg/ml). Entacapone decreases the peripheral degradation of levodopa and thus increases it's bioavailability.^11^ Similarly to LCIG treatment, a constant infusion of LECIG is delivered with portable infusion pump directly to the proximal jejunum via surgically installed percutaneous endoscopic trans‐gastric jejunostomy (PEG‐J) tube.^12^

The effectiveness of LECIG to motor symptoms appears similar to LCIG treatment observed in one 48 h crossover study.^10^ An existing study has suggested that patients prefer LECIG to LCIG because of the smaller size and weight of the pump system (227 g vs. 550 g with a full cassette, respectively).^13^ Another possible benefit of LECIG compared to LCIG is reduced daily dose of levodopa due to increased bioavailability, as higher doses of levodopa have been associated to increased risk of polyneuropathy.^12^, ^14^ Furthermore, it has been suggested that entacapone may have a protective role against polyneuropathy.^15^

The LECIG treatment was first introduced in Sweden in 2019 and the first patient in Finland initiated the treatment in August 2020. Currently there are only a few published studies of LECIG treatment. Here we report our clinical findings including adverse events, changes in medication and early discontinuations on patients treated with LECIG infusion in Helsinki University Hospital (HUS).

## Methods

In this retrospective longitudinal observational study, patient records of 30 consecutive advanced PD patients, who received LECIG treatment between August 2020 and December 2022, in HUS, were reviewed. All data were extracted from the data system of HUS. Following time points were used for data collection: baseline before LECIG initiation, hospital discharge with LECIG and six months after initiation.

For LECIG patient selection, in our center, we use the same protocol as for LCIG candidates, with the exception, that evidence of suitability of entacapone to the patient must exist in history, or be tested with oral entacapone at the minimum for two months, prior to LECIG initiation. Inclusion criteria are advanced PD with motor fluctuations and/or disabling dyskinesia poorly controlled with oral medication. Exclusion criteria are advanced dementia, which is defined as Mini Mental State Examination (MMSE) score <20,^16^ clinical suspicion of atypical parkinsonism, insufficient responsiveness to levodopa, adverse events related to oral entacapone, and ongoing psychosis not responsive to antipsychotic medication. Our clinical protocol is thoroughly described in a recently published article.^17^ If both LCIG and LECIG are suitable for the patient, we let the patient choose the option he or she prefers.

In many centers, levodopa responsiveness, suitability and titration of LCIG treatment, before PEG‐J tube installation, is confirmed by levodopa‐carbidopa infusion via temporary naso‐jejunal tube (NJT).^18^, ^19^, ^20^ However, NJT may cause discomfort to the patient, is time consuming and susceptible to complications.^21^ Consequently, some centers have also used direct PEG‐J tube initiation.^9^, ^22^, ^23^ This alternative protocol started at our center in year 2015 first with LCIG patients, and is used today also with LECIG patients. The choice between direct initiation and NJT trial is made on a case‐by‐case basis on discretion of the movement disorder specialists of our unit. Reasons for preceding NJT trial are, for example, cognitive decline and living alone. We perform a levodopa challenge test (LCT) to confirm levodopa responsiveness to all patients either before direct initiation or during the NJT test phase.^24^

All information was collected from the patient records including the following baseline data: age, gender, medication, levodopa equivalent daily dose (LEDD), the clinical state of PD evaluated by Hoehn and Yahr score (H&Y), living situation and the duration of PD (from date of diagnosis to the initiation of LECIG). LEDD was calculated according to standard procedures.^25^ If not directly stated in the patient records, H&Y was evaluated from the data available.

Homocysteine, folate, B6‐ and B12‐vitamin levels and weight were measured before the initiation of treatment and at six months. Adverse events, discontinuations of treatment, and medication, including LEDD, were collected from the follow‐up.

Statistical calculations were generated using IBM SPSS Statistics 28 (International Business Machines Corporation, Endicott, NY). Paired sample t test was used to evaluate changes in weight, LEDD, and levodopa and entacapone doses during the follow‐up. All P values reported are 2‐tailed and a *P* ≤ 0.05 was considered statistically significant.

The study had multiple objectives. We evaluated the changes in medication, adverse events and early discontinuations of LECIG treatment in real life clinical practice.

## Results

**FIG. 1:**
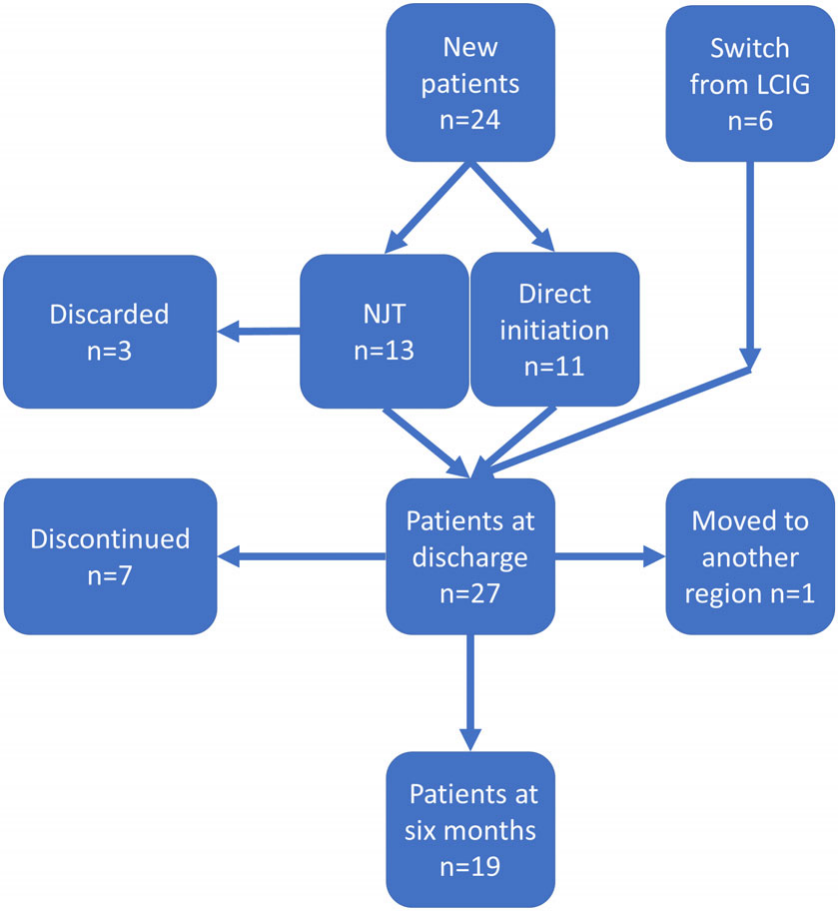
Flowchart of the 30 study patients. NJT, nasojejunal tube; LCIG, levodopa‐carbidopa intestinal gel.

The epidemiological data of the 30 study patients is shown in Table 1. LECIG treatment was initiated via PEG‐J tube either after the NJT test phase, directly after a positive LCT, or directly switching from LCIG to LECIG. Three patients were discarded during the NJT test phase. Of the 27 patients who initiated LECIG treatment via PEG‐J tube, seven (26%) discontinued the treatment before six‐month follow‐up and one moved to another region and was thus lost to follow‐up. Consequently, 19 patients reached the six‐month follow‐up. Flow chart of the patients is shown in Figure 1. The mean (range) exposure to LECIG was 149 (5–183) days while the total exposure was 11 years.

**TABLE 1:** Epidemiological data

Gender (female), *n* (%)	18 (60)
Age (yr), mean (SD)	72 (6.3)
Disease duration (yr), mean (SD)	13 (4.7)
Living situation	
Alone, *n* (%)	11 (37)
Spouse, *n* (%)	17 (57)
Institution, *n* (%)	2 (6.7)
HY, *n* (%)	
2	2 (6.7)
3	15 (50)
4	13 (43)
Medication, *n* (%)	
Antidepressant	6 (20)
Antipsychotic	7 (23)
Anti‐dementia	3 (10)

*Note*: Baseline features of the 30 study patients.

Abbreviations: SD, Standard deviation; HY, Hoehn et Yahr index.

All patients were eligible for both LCIG and LECIG treatments, but chose LECIG due to its smaller pump system. The six switches from LCIG to LECIG were uniformly executed for the same reason. The mean age (SD) and disease duration of the six patients, who switched from LCIG to LECIG, were 77 (4.0) and 13 (4.9) years, respectively. Before switching to LECIG, they had used LCIG for 35 months on average (range 7–65 months).

All patients used LECIG for 16 h daily during the follow‐up. The anti‐Parkinson medication used by the patients during the LECIG treatment is shown in Table 2. All patients had tolerated oral entacapone without major side effects before LECIG initiation and 83% were currently using oral entacapone at baseline prior to LECIG initiation.

**TABLE 2:** Anti‐Parkinson medication during LECIG treatment

	Before initiation	At discharge	At 6 months
*N*	30	27	19
LECIG morning dose (ml), mean (SD)	‐	8.3 (3.0)	9.1 (2.7)
LECIG daily infusion (ml/h), mean (SD)	‐	2.3 (0.79)	2.5 (0.70)
MAO‐B inhibitor, *n* (%)	11 (37)	4 (15)	4 (21)
Dopamine agonist, *n* (%)	19 (63)	12 (44)	9 (47)
Controlled release levodopa, *n* (%)	16 (53)	21 (79)	16 (84)

*Note*: Changes in anti‐Parkinson medication used by the patients during LECIG treatment.

Abbreviations: SD, Standard deviation; LECIG, levodopa‐entacapone‐carbidopa intestinal gel; COMT, catechol‐O‐methyltransferase; MAO‐B, monoamine oxidase‐B.

**FIG. 2:**
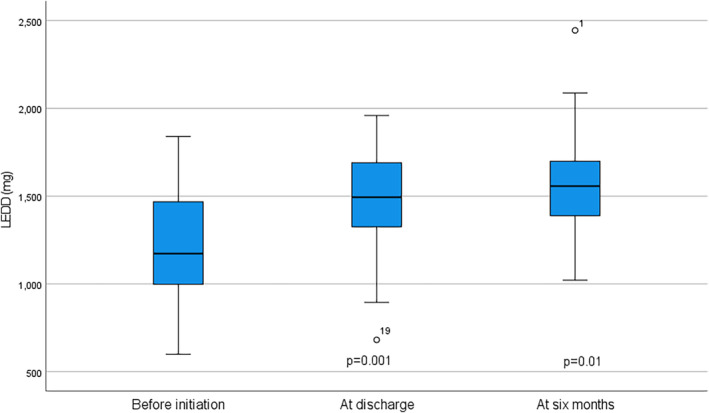
The changes in LEDD of 19 LECIG‐patients from baseline before initiation to six months with treatment. P values are calculated for differences between each time point and the previous time point. LEDD, levodopa equivalent daily dose; LECIG, levodopa‐entacapone‐carbidopa intestinal gel.

During the follow‐up, there was a significant increase in LEDD from baseline before LECIG treatment to six months with treatment (1230 mg vs. 1570 mg, *P* = 0.001), as depicted in Figure 2. Similarly, there was a rise in the daily levodopa dose (891 mg vs. 1096 mg, *P* = 0.03) during the same follow‐up period. However, mean entacapone dose did not change (1075 mg vs. 1018 mg, *P* = 0.64, *n* = 16) between baseline before LECIG initiation (oral entacapone) and six‐month follow‐up (LECIG‐infusion). At discharge 19% of patients used LECIG monotherapy without concomitant anti‐Parkinson medications. At six months this figure was 16%.

Three patients were discarded during the NJT test phase; two because of neuropsychiatric problems (delirium and hallucinations) and one because of difficulties using the pump system. Seven (26%) patients discontinued the treatment during the first six months. One patient died of a reason unrelated to LECIG treatment. Two patients discontinued the treatment due to difficulty in finding a suitable infusion rate, and two due to neuropsychiatric problems. One patient, who had had problems in finding optimal infusion rate at the initiation of LECIG treatment, later developed rhabdomyolysis due to severe dyskinesia and discontinued the treatment. Of the patients who switched from LCIG to LECIG treatment, only one discontinued the treatment during the follow‐up (due to neuropsychiatric problems), while the other five patients continued the treatment beyond six months.

The weights of three patients, who had difficulties in finding suitable infusion rates, were 56, 50 and 54 kg when the average baseline weight of the patients who reached the six‐month follow‐up was 67 kg (min 45, max 107). On average, there was no significant change in weight between baseline and six months (67 kg vs. 66 kg, *P* = 0.43). However, there were two cases of significant weight loss, of which the other was considered as treatment related.

There were 11 tube replacements during the follow‐up resulting in 1.0 replacements per patient year. Reasons for discontinuation and complications are shown in Table 3.

**TABLE 3:** Discontinuations and complications

Discontinuations	*N*
Total	7
Difficulty in finding suitable infusion rate	2
Neuropsychiatric problems	2
Difficulty in using the device	1
Rhabdomyolysis	1
Death	1
Inner tube replacements	
Total	10
Dislocation	7
Blockage	3
PEG tube replacements	1
Other complications	
Polyneuropathy	0
Weight loss	2
Major infections	0
Device malfunction	1

*Note*: Complications, reasons for discontinuations and reasons for tube replacements of the 27 levodopa‐entacapone‐carbidopa intestinal gel patients during first six months of the treatment.

Abbreviation: PEG, percutaneous endoscopic gastrostomy.

The vitamin and homocysteine levels measured before the initiation of the treatment and at six‐month follow‐up are shown in Table 4.

**TABLE 4:** Changes in laboratory parameters

	Before initiation	At six months
Homocysteine (umol/l), mean (SD)	18 (6.3)	15 (3.9)
Folate (nmol/l), mean (SD)	19 (8.7)	30 (14)
B6‐vitamin (nmol/l), mean (SD)	106 (104)	111 (74)
B12‐vitamin (pmol/l), mean (SD)	105 (36)	105 (44)

*Note*: Changes in laboratory parameters measured at baseline before the initiation of levodopa‐entacapone‐carbidopa‐infusion‐gel treatment and at six months during the treatment. *N* = 16.

Abbreviation: SD, Standard Deviation.

## Discussion

Comparable with an existing study, all patients eligible for intestinal levodopa‐infusion treatment chose LECIG over LCIG due to smaller pump size.^13^ Similarly, the main reason for patients to switch from LCIG to LECIG was the pump size.

Mean LEDD rose significantly during the first six months of treatment (1230 mg vs. 1570 mg, *P* = 0.001) as did the daily levodopa dose. In comparison to studies considering LCIG, GLORIA registry, where mean baseline LEDD was 1319 mg, shows changes of similar magnitude at six months (1653 mg in LCIG monotherapy and 2013 mg in combination therapy).^18^ However, in recent DUOGLOBE study, LEDD remains relatively stable from three months to six months, and the mean daily dose of LCIG remains fairly stable from Day 1 (1241.2 mg) through month 36 (1365.4 mg).^26^ This difference may be explained by different starting points of the follow‐up, since according to the results of GLORIA registry, the largest increase in LEDD happens at the initiation of treatment (from baseline to Day 1). Our hypothesis is that as in LCIG treatment, patients tolerate higher doses of levodopa due to stable levodopa plasma concentration produced by constant infusion.^17^

The adverse events encountered during the study were similar to those reported with LCIG treatment.^18^, ^27^ In addition, there was one case of rhabdomyolysis due to excessive dyskinesia. This rare complication of dopaminergic treatment has also been previously described with LCIG patients.^28^ The incidence of tube replacements was 1.0 per patient year which is higher than expected from our earlier study on LCIG patients in which the incidence was 0.38 per patient year.^17^ However, the length of follow‐up was different and the study population in the present study was, on average, older and had longer disease duration than the patients in our earlier study considering LCIG. Of note, to address the tube issues in the future, the novel T‐port appears to have minimal risk of internal tube disconnection compared with existing PEG‐J systems.^29^

Patients who did not tolerate the oral COMT inhibitor were excluded from the LECIG treatment. Earlier study has shown that patients who suffered from diarrhea with oral entacapone, also developed it during LECIG treatment.^13^ Cases of liver damage caused by COMT inhibitor tolcapone have raised concerns considering other COMT inhibitors.^30^ However, entacapone has not been shown to cause liver damage in clinical trials or post‐marketing surveillance of tablet formulations.^31^ Furthermore, in the present study, the average entacapone dose did not change during the follow‐up for those who were using oral entacapone at baseline before LECIG initiation. Yet, studies considering liver function with LECIG treatment are lacking and liver function test monitoring should be considered especially with patients with high doses of LECIG.

The discontinuation rate during the first six months of LECIG treatment was 26%. Similarly, in a recent study from Sweden, where median LECIG treatment duration was 305 days, the discontinuation rate was 25%.^13^ These rates appear higher than those reported with LCIG. For example, Sensi et al have reported 9.5% discontinuation rate during the first treatment year in their large cohort of 905 LCIG patients.^32^ Comparably, in our center, discontinuation rate in a group of 103 LCIG patients was 15% during the first year.^17^ Further research is required with a larger group of patients to examine this matter thoroughly.

Two patients discontinued the treatment because of difficulties finding suitable infusion rate. In addition, the patient who eventually developed rhabdomyolysis due to dyskinesia had also had problems in determining the optimal LECIG infusion dose when initiating the treatment. As an example, patient number 11 had troublesome dyskinesia with infusion rate of 2.6 ml/h and went into OFF‐state with infusion rate of 2.5 ml/h. Interestingly, these three patients weighed approximately 10 kg less than the average patient who reached the six‐month follow‐up. Patients with low body weight might benefit from an option of smaller increments in infusion rate as the currently smallest possible increment (0.1 ml/h) may be too large for some of them.

Polyneuropathy is associated to levodopa and especially to LCIG treatment with a possible causative role of levodopa metabolite homocysteine.^14^ It is suggested that levodopa methylation by COMT increases the homocysteine levels which among other metabolites may cause polyneuropathy.^15^ Consequently, it is proposed that the COMT inhibitor may have a protective role against polyneuropathy.^15^ In the present study, no clinically diagnosed cases of polyneuropathy appeared during the first six months of LECIG treatment. Furthermore, no increase was seen in homocysteine levels. However, as a confounding factor, many patients used over the counter vitamin supplements. Furthermore, routine ENMG recordings were not done during the follow‐up.

The present study has some strengths and limitations. The retrospective design of the study leads to some missing data and the small sample size prevents certain statistical analyses. However, considering the scarcity of scientific knowledge of LECIG treatment, the current study offers valuable information on this novel treatment option in real life clinical practice.

In conclusion, LEDD and daily levodopa dose appear to increase after the initiation of LECIG similarly to LCIG treatment. As expected, adverse events encountered with LECIG are similar to those with LCIG. The early discontinuation rate in LECIG appears high when compared to studies considering LCIG. Apart from the small pump system, which the patients prefer, no clear advantage of LECIG compared to LCIG can be established. However, long‐term data are lacking. Further research with larger patient groups and longer follow‐up is needed to examine the safety and efficacy of LECIG treatment. Low body weight may pose a risk to a narrow therapeutic window of LECIG treatment which may lead to discontinuation of the treatment. To address this, smaller increments could be considered as an option to enable these patients to continue with LECIG treatment.

## Author Roles

(1) Research project: A. Conception, B. Organization, C. Execution; (2) Statistical Analysis: A. Design, B. Execution, C. Review and Critique; (3) Manuscript: A. Writing of the first draft, B. Review and Critique.

V.V.: 1C, 2A, 2B, 3A

M.T.: 2A, 2C, 3B

P.K.A.M.: 1B, 3B

K.M.: 1B, 3B

E.R.J.: 1B, 3B

U.M.: 1B, 3B

P.E.: 1A, 1B, 2C, 3B

## Disclosures

**Ethical Compliance Statement:** Clinical research permit was approved by the Department of Neurology of Helsinki University Hospital. According to the Finnish law, retrospective research using hospital medical files does not require informed consent from the study subjects. We confirm that we have read the Journal's position on issues involved in ethical publication and affirm that this work is consistent with those guidelines.

**Funding Sources and Conflict of Interest:** VV has received a grant from the Finnish Parkinson Foundation and Helsinki University Hospital. No conflict of interest. MT has received a grant from Helsinki University Hospital. No conflict of interest. PKAM has no conflict of interest to report. KM has no conflict of interest to report. ERJ has no conflict of interest to report. UM has no conflict of interest to report. PE has no conflict of interest to report.

**Financial Disclosures for the Previous 12 Months:** VV has no additional disclosures to report. MT has no additional disclosures to report. PKAM has received lecture fees from AbbVie. KM has no additional disclosures to report. ERJ has received consultation fees from NordicInfu Care. UM has no additional disclosures to report. PE is PI in Finland: International Adroit‐study (Abbott DBS Registry of Outcomes for Indications over Time). 2021‐: Organized by Abbott. He is consulting Neurologist of Patient Insurance Centre and a member of Advisory boards of Abbvie, Boston Scientific, and NordicInfu Care. He has received consulting fees from NordicInfu Care AB and Abbvie, and has received lecture fees from Abbott, Abbvie and NordicInfu Care.
